# The Ambulatory Pediatric Association Fellowship in Pediatric Environmental Health: A 5-Year Assessment

**DOI:** 10.1289/ehp.10015

**Published:** 2007-06-28

**Authors:** Philip J. Landrigan, Alan D. Woolf, Ben Gitterman, Bruce Lanphear, Joel Forman, Catherine Karr, Erin L. Moshier, James Godbold, Ellen Crain

**Affiliations:** 1 Department of Community and Preventive Medicine, Mount Sinai School of Medicine, New York, New York, USA; 2 Department of Pediatrics, Mount Sinai School of Medicine, New York, New York, USA; 3 Department of Pediatrics, Children’s Hospital, Boston and Harvard Medical School, Boston, Massachusetts, USA; 4 Department of Pediatrics, George Washington University School of Medicine, Washington, DC, USA; 5 Department of General Pediatrics, Cincinnati Children’s Hospital Medical Center, Cincinnati, Ohio, USA; 6 Department of Pediatrics, University of Washington School of Medicine, Seattle, Washington, USA;; 7 Department of Pediatrics (Emergency Medicine), Jacobi Medical Center, Albert Einstein College of Medicine, Bronx, New York, USA

**Keywords:** community pediatrics, environmental medicine, environmental pediatrics, fellowship training, medical education

## Abstract

**Background:**

Evidence is mounting that environmental exposures contribute to causation of disease in children. Yet few pediatricians are trained to diagnose, treat, or prevent disease of environmental origin.

**Objectives:**

To develop a cadre of future leaders in pediatric environmental health (PEH), the Ambulatory Pediatric Association (APA) launched a new 3-year fellowship in 2001—the world’s first formal training program in PEH. Sites were established at Boston Children’s Hospital, Mount Sinai School of Medicine, George Washington University, University of Cincinnati, and University of Washington. Fellows are trained in epidemiology, biostatistics, toxicology, risk assessment, and preventive medicine. They gain clinical experience in environmental pediatrics and mentored training in clinical research, policy development, and evidence-based advocacy. Thirteen fellows have graduated. Two sites have secured follow-on federal funding to enable them to continue PEH training.

**Discussion:**

To assess objectively the program’s success in preparing fellows for leadership careers in PEH, we conducted a mailed survey in 2006 with follow-up in 2007.

**Conclusions:**

Fifteen (88%) of 17 fellows and graduates participated; program directors provided information on the remaining two. Nine graduates are pursuing full-time academic careers, and two have leadership positions in governmental and environmental organizations. Ten have published one or more first-authored papers. Seven graduates are principal investigators on federal or foundation grants. The strongest predictors of academic success are remaining affiliated with the fellowship training site and devoting < 20% of fellowship time to clinical practice.

**Conclusion:**

The APA fellowship program is proving successful in preparing pediatricians for leadership careers in PEH.

Causes of death and disease among children in the United States have changed profoundly in the past 100 years (Hoyert et al. 2006). Infant mortality has declined by 90%. Life expectancy at birth has doubled. The major diseases of American children today are a group of chronic conditions that are termed the “new pediatric morbidity” (Haggerty and Rothman 1975):

- Asthma, the leading cause of hospitalization and school absenteeism, has more than doubled in incidence since the 1970s (Akinbami and Schoendorf 2002).
- Birth defects are the leading cause of infant death and subsequent morbidity. Incidence rates of certain birth defects, such as hypospadias, have increased sharply [Centers for Disease Control and Prevention (CDC) 2006; Paulozzi et al. 1997].
- Neurodevelopmental disorders—autism, attention deficit/hyperactivity disorder, learning disabilities, dyslexia, and mental retardation—affect 5–10% of the 4 million babies born in the United States each year. Incidence appears to be increasing (Bertrand et al. 2001; Boyle and Cordero 2005; Honeycutt et al. 2003; LeFever et al. 1999; Zito et al. 2000).
- Childhood and young adult cancers—acute leukemias, primary brain cancer and testicular cancer—have seen incidence rates increase steadily over the past three decades, despite improved treatments that have greatly reduced mortality (Devesa et al. 1995; National Cancer Institute 2007).
- Obesity and type 2 diabetes have become epidemic (Dietz 2004).
- Preterm birth, affecting > 12% of U.S. children, has increased in incidence by 27% since 1981 (Hoyert et al. 2006).

## Mounting evidence for environmental causation of disease

Clinical and epidemiologic evidence gathered over the past half-century has shown that environmental factors—toxic chemicals [CDC 2005; Commission for Environmental Cooperation 2006; Houlihan et al. 2005; National Research Council (NRC) 1993; U.S. Environmental Protection Agency (EPA) 1998] and the modern built environment (Galvez et al. 2003; Giles-Corti et al. 2003; Jackson 2003)—can contribute to the causation of disease in children. Infants and children are especially sensitive to environmental exposures because of their disproportionately heavy exposures and the biological susceptibility that is an inherent consequence of early development (NRC 1993). Documented examples of disease of environmental origin in children include phocomelia in infants exposed *in utero* to thalidomide (Lenz and Knapp 1962); adeno-carcinoma of the vagina in girls exposed prenatally to diethylstilbestrol (Herbst et al. 1981); increased incidence of childhood asthma and increased frequency of acute asthmatic exacerbations in children exposed to particulate air pollution and secondhand cigarette smoke (Friedman et al. 2001; Gauderman et al. 2004; Salam et al. 2004; Suh et al. 2000); increased incidence of sudden infant death syndrome in babies exposed to particulate air pollution (Woodruff et al. 1997); neurocognitive impairment and disruption of behavior among children exposed *in utero* or early in postnatal life to lead (Canfield et al. 2003; Needleman et al. 1979), polychlorinated biphenyls (Jacobson and Jacobson 1996), methylmercury (Budtz-Jorgensen et al. 2002; Grandjean et al. 1997), and ethyl alcohol (Barr and Streissguth 2001); and small head circumference and slowed learning in children exposed prenatally to organophosphate pesticides (Berkowitz et al. 2004; Rauh et al. 2006).

Beyond childhood, incidence rates of chronic neurodegenerative diseases of adult life such as Parkinson’s disease and dementia have increased markedly in recent decades. These trends raise the possibility that exposures in early life may act as triggers of later illness, perhaps by reducing the numbers of cells in essential regions of the brain to below the level needed to maintain function in the face of advancing age (Cory-Slechta et al. 2005).

## Current state of environmental pediatrics

Despite the growing body of knowledge about the importance of the environment to children’s health, few pediatricians have training or experience in the diagnosis, treatment, or prevention of diseases of environmental origin (Hu and Woolf 2003). Pediatricians report that they frequently encounter diseases that appear to be initiated by environmental factors, but most report discomfort and lack of information in dealing with these conditions (Kilpatrick et al. 2002; Trasande et al. 2006; Woolf and Cimino 2001). A root cause is that the curriculum of the average U.S. medical school devotes only 6 hours of teaching time to topics in environmental health (Burstein and Levy 1994). Of 125 medical schools whose curricula are listed in the Curriculum Directory of the Association of American Medical Colleges, only two report a required course in environmental medicine (Association of American Medical Colleges 2006). Only a handful of academic health centers in the United States have trained faculty or clinical referral centers in pediatric environmental health (PEH). To improve this situation, the Institute of Medicine (IOM) has recommended that education in environmental health become an integral component of medical education and has produced a set of competency-based learning objectives in environmental health (IOM 1995).

Recent developments at the federal level—most notably the establisment by CDC/ Agency for Toxic Substances and Disease Registry (ATSDR) of 11 pediatric environmental health specialty units (PEHSUs) across the United States, and the impending launch of the National Children’s Study (Landrigan et al. 2006)—will increase further the national need for a scientific workforce trained in PEH.

## The APA fellowship in PEH

To develop the careers of future leaders in PEH and thus to create the core of a national scientific work-force, the Ambulatory Pediatric Association (APA) established a unique new postresidency Fellowship Program in PEH in 2001 at selected academic health centers. This fellowship is the first formal training program in PEH in the United States and the world.

The intent of the PEH fellowship is to produce a highly skilled cadre of physician-scientists who will have interdisciplinary training in pediatrics and prevention, expertise in epidemiology, biostatistics, and risk assessment, and a deep fund of knowledge about the impacts of the environment on human development and child health. These clinician-scientists will be essential to meet the challenge of understanding, treating, and preventing the “new pediatric morbidities.”

The purpose of this report is to describe the genesis and structure of the PEH fellowship program, to review its early history, to present the results of a preliminary evaluation of fellows’ career progress, and to assess objectively the success of the program in producing future leaders in environmental pediatrics. Additionally, we discuss prospects for the future academic and professional development of the field of PEH, including issues of funding and credentialing.

## Program Description

### Goals and objectives

The goal of the APA Fellowship Program in PEH is to train a select cadre of pediatricians who will become the next generation of physician-scientists and academic leaders in pediatric environmental medicine. To guide development of the program, the APA formed a Fellowship Oversight Committee that is composed of academic leaders in PEH from across the United States and that reports directly to the APA Board of Directors. In 2001, this committee secured two founding grants on behalf of the APA from the New York Community Trust and the Educational Foundation of America to support the launch of the PEH fellowship program.

### Program initiation

To announce the program, the oversight committee developed a request for proposals that was sent to the chairs of all Accreditation Council for Graduate Medical Education–accredited Departments of Pediatrics at U.S. medical schools. Eight programs returned applications, and these applications were reviewed and ranked by a group of four nationally recognized experts in PEH and the then-president of the APA. The oversight committee then conducted a second-tier review. Through this process, three training sites were selected: Harvard Medical School/ Boston Children’s Hospital, Mount Sinai School of Medicine, and George Washington University Medical Center. Additional training sites were established at the University of Cincinnati [supported by a National Research Service Award from the Health Resources and Services Administration (HRSA)] and at the University of Washington (supported by a training grant in environmental epidemiology from the National Institute of Environmental Health Sciences). The program was announced nationally, and pediatricians across the United States were invited to apply for fellowship positions.

### Training competencies

To guide the development of curricula and the training of fellows, the Fellowship Oversight Committee developed a set of competency-based training objectives in PEH (Etzel et al. 2003). These competencies were unanimously approved by the Committee on Environmental Health of the American Academy of Pediatrics.

### Program architecture

Because the American Board of Pediatrics requires that all fellowship programs be 3 years in duration, the PEH fellowship is designed as an intensive 3-year academic experience. It is comprised of didactic training leading to an MPH, MS, or PhD degree (40% of time), mentored research (40% of time), and training in clinical environmental medicine and community advocacy (20% of time). All fellows are expected to write a thesis. Curricula are coordinated and approved by the Fellowship Oversight Committee.

Each training site has organized an internal advisory board that works with the program director to oversee the selection of faculty mentors, the recruitment and selection of new fellows, the evaluation of each fellow’s progress, and the assessment of overall program quality. Selection of new fellows of high academic caliber and strong leadership potential is a key responsibility of the internal advisory board, and to secure the best available candidates, program advertisements are distributed widely, and fellows are chosen through a competitive, peer-reviewed process. Each site also has established an external advisory committee. Each program director reports annually to the APA Fellowship Oversight Committee.

### Didactic curriculum

All fellows receive didactic training in epidemiology, biostatistics, study design, data management, data analysis, environmental medicine, toxicology, exposure assessment, research ethics, policy analysis, and community outreach. They receive instruction in grant writing and practical guidance in career building and in obtaining research funding. The bulk of the formal didactic training is completed in the first 2 years of fellowship.

### Research experience

A key component of the fellowship is a flexible, individually tailored, closely mentored research experience. This experience is the principal vehicle through which fellows gain the skills and experience that they need to move from traineeship to independent investigator status.

The mentored research experience begins early in the fellowship in July–September of the first year. During these initial months, the program director and co-directors introduce each fellow broadly to faculty members, and they encourage fellows to meet with multiple faculty to explore their ideas and to identify a primary mentor and advisors. By the end of September of the first year, each fellow is expected, in consultation with the program director and the internal advisory committee, to have chosen a mentor and developed the preliminary outline of a research proposal. In some programs, the mentor is selected before a candidate is offered a position. By the end of December of the first year, each fellow is expected to produce a preliminary research proposal. Each fellow has weekly or bi-weekly meetings throughout this year with his or her mentor and advisors to ensure that the fellow’s time is properly protected and that the mentored research project is on schedule. In the latter half of the first year and at an accelerating pace in the second year, the fellow begins to implement the research protocol. The fellow collects research data, designs and implements a data management plan, begins to analyze the data with assistance from the research mentor(s) and program faculty, and prepares an abstract and presentation of the research.

Each fellow is expected by the end of the 3-year fellowship program to have completed the research project and prepared a first-authored research manuscript suitable for submission to a peer-reviewed journal. The fellows are also expected to submit an abstract of their research for presentation at the annual meeting of the APA.

### Clinical training

Each fellow is expected to participate in supervised clinical activities in environmental pediatrics during the fellowship. At Mount Sinai and at the University of Washington, this requirement is met through service in the CDC/ATSDR-supported PEHSUs. At the University of Cincinnati, it is met by one half-day of service each week in the Lead Treatment Clinic or by another relevant clinical experience. At Boston Children’s Hospital, PEH fellows fulfill the clinical requirement by performing clinical work in the Pediatric Environmental Health Center, which is linked closely to the Massachusetts/ Rhode Island Poison Control Center (Shannon et al. 2003).

### Advocacy training

Acquisition of skill in evidence-based advocacy and in the translation of medical findings to public policy is an essential component of the APA fellowship program. At Boston Children’s Hospital, fellows learn the skills of evidence-based advocacy through collaboration with the Harvard School of Public Health’s Center for Children’s Environmental Health; this center studies exposures to metals among Native American children living near former mining sites. At Mount Sinai, fellows had a unique opportunity to perform evidence-based advocacy in the aftermath of the attacks on the World Trade Center. They provided guidance to community groups in lower Manhattan, to the New York City Department of Health, and to the Board of Education on protecting children from the toxic chemicals released into the environment by the destruction of the towers. At the University of Washington, fellows gain experience in advocacy through interaction with the Region 10 PEHSU.

### Evaluation

To assess each fellow’s progress, a rigorous evaluation process has been established at each training site. Each fellow provides a written assessment of progress every 6 months and meets with the program director at least every 3 months. Key mentors complete annual written evaluations of each fellow’s performance. Each fellow’s program is continuously retailored as required.

### Fellows’ retreat

Each year the APA convenes a 3-day retreat of fellows and faculty from all of the training sites, modeled after the annual retreat of the Robert Wood Johnson Clinical Scholars’ Program. At these retreats, fellows and faculty have an opportunity to present and review each other’s research, to hear lectures and participate in roundtables convened by leading scientists, physicians, and policy makers, and to discuss future development of PEH.

### Conclusion of funding

Funding for the APA Fellowship in PEH has now concluded, because support for the program was based on two nonrenewable foundation grants. APA continues to host the annual fellows' retreat and to encourage academic health centers to acquire independent funding to further sustain training in PEH. To date, two sites—the Mount Sinai School of Medicine and the University of Cincinnati—have secured independent funding and are continuing to train fellows.

## Methodology

### Questionnaire development

To assess the impact of the APA fellowship program on fellows’ careers and research productivity, we developed a self-administered mail questionnaire for current fellows and program graduates. This instrument was designed to gather information on the demographics of trainees, their duration of training, advanced degrees obtained through the fellowship, and relative time allocated to course work, research, and other activities during the fellowship. It was based on a survey of fellows in general pediatrics supported by HRSA/National Research Service Award (NRSA) training grants conducted by Steiner et al. (2002). Emphasis was placed on ascertaining aspects of the fellowship and of the mentoring process that were the most important determinants of future academic success (Curtis et al. 1992; NRC 2000; Steiner et al. 2000; Zakowski et al. 1998).

Participants were asked to describe characteristics of their current professional position, including their employer, academic affiliation, faculty rank, current time allocation and hours worked per week, their role on current research projects, and their number of first-authored and co-authored publications since the beginning of the fellowship.

The survey was administered by mail, with two follow-up mailings at intervals of 3 weeks, followed by attempted phone or email contacts with individuals who had not replied. The initial mailing was sent out in 2006, and a follow-up was conducted by telephone in 2007.

This survey was granted exempt status by the Office of Grants & Contracts of the Mount Sinai School of Medicine.

### Participants

All current APA PEH fellows and graduates of the program were eligible for the survey. We identified program participants using training records and rosters from the training sites.

### Measures of research productivity

We defined two outcome markers to assess the research productivity of program graduates, based on similar measures used in prior studies (Lee et al. 1991; Levey et al. 1988; Rodgers and Scherbenske 1990): self-reported publication of one or more papers per year as first author or co-author since the beginning of fellowship, and self-reported acquisition of peer-reviewed funding as principal investigator from any federal or nonfederal source. We examined the influence of fellowship characteristics on research productivity, with particular emphasis on examining how time allocation within the fellowship—academics versus clinical practice versus protected research time—influenced success.

### Data analysis

We performed two-group comparisons using the Mann-Whitney *U-*test.

## Results

### Survey participation

A total of 17 fellows have entered the APA Fellowship in PEH since its inception in 2001—six at the Mount Sinai School of Medicine, four at Harvard Medical School/Boston Children’s Hospital, one at George Washington University, five at the University of Cincinnati, and one at the University of Washington. Thirteen of these 17 fellows have graduated from the program, and 4 are still in training.

Fifteen (88%) of the 17 fellows and graduates completed the self-administered mail survey sent out in 2006 and provided follow-up information in the telephone survey in 2007. Among graduates, the participation rate was 11 of 13 (85%), and among current fellows it was 8 of 8 (100%). Program directors provided limited information on the two graduates who did not respond.

### Mentoring

All fellows, past and current, reported that they had a mentor during fellowship, that the mentor was “particularly influential,” and that they continued even after completion of the fellowship to receive guidance from the mentor. Eight of the 11 graduate responders reported that they wrote a grant proposal during their fellowship.

### Career trajectory

Of the 13 graduates, nine are pursuing academic careers as full-time faculty in academic health centers, one is a senior epidemiologist in a major metropolitan health department, and one is a scientist with a national environmental organization. The remaining two are in private practice settings. Thus the overall success of the fellowship program in producing graduates who are currently on track to become future leaders in PEH is 85%.

### Academic success

Ten graduates of the fellowship reported having published one or more first-authored papers (mean 2.1; range 1–3). Seven have also co-authored papers (mean 3; range 0–8). Four graduates are principal investigators on federal (2) or foundation (2) grants, and one current fellow is principal investigator on a federal grant. The strongest predictors of academic success are remaining affiliated with the fellowship training site (*p* = 0.04) and devoting < 20% fellowship time to clinical practice (*p* = 0.003) (Tables 1–4).

## Discussion

The APA fellowship program in PEH is this nation’s and the world’s first formal training effort in PEH. Previously, pediatricians who wished to acquire training in environmental medicine were required to approach the field though a variety of self-initiated pathways, such as residency or fellowship training in occupational medicine, epidemiology, or toxicology; service in a poison control center; or completion of a research apprenticeship.

Today, 6 years after inception of the APA fellowship in PEH, program graduates are beginning to populate academic health centers. Our survey found that 9 of 13 program graduates are pursuing full-time academic careers, and that another two have taken scientific leadership positions in governmental and national environmental organizations. These graduates are actively publishing, and they have secured peer-reviewed grant funding. Although these early findings are based on a small number of relatively recent graduates, they are consistent with the early academic success of graduates of the Robert Wood Johnson Clinical Scholars Program and of the NRSA-funded primary care training grants (Shannon et al. 2003). They attest to the robustness of the program and argue the need and feasibility of further expansion.

Two major challenges confront the future development of PEH and the graduates of this fellowship program: funding and credentialing.

Funding is a problem at the federal level in this era of declining support for biomedical research. The percentage of research grant and career development grant proposals that are funded is in sharp decline, and it is especially difficult for young investigators to secure funding. Support for the national program of Children’s Environmental Health and Disease Prevention Research Centers is under review. The budget for the National Children’s Study had been in jeopardy, but appears recently to have been rescued by the strong and decisive action of the U.S. Congress. Funding is also a problem in the private sector. Most reimbursement schedules do not adequately cover providers for the detailed history taking or the specialized investigations that are needed to evaluate the environmental causes of disease in children. A further impediment is that payers (government, private insurers, health maintenance organizations) will not currently cover the costs of recommended environmental remediation within individual families/households. As an example, low-income families often cannot afford prescribed dust remediation for prevention of asthmatic reactions or paint removal for lead abatement. New funding sources and new partnerships will be required to address these problems. On a positive note, two of the training sites that participated in the APA fellowship—the Mount Sinai School of Medicine and the University of Cincinnati—have now secured federal training grants to continue their training programs in PEH; the University of Cincinnati is receiving continuing support from a National Research Service Award from HRSA, and Mount Sinai has been awarded a T32 training grant in PEH by the National Institute of Child Health and Human Development.

Credentialing issues need to be addressed to clarify career pathways for graduates of the PEH fellowship program. Clarification of these issues may also assist with problems pertaining to reimbursement for specialty-based services in PEH. One option is to seek sub-specialty board certification in PEH from the American Board of Medical Specialties. This will be a multiyear effort and will require careful specification of the boundaries and special competencies that distinguish PEH from such other, already established specialties as occupational and environmental medicine, and the medical toxicology subboard within the boards of preventive medicine, pediatrics, and emergency medicine. A second option is subspecialty certification in partnership with one or more existing specialties. A third approach is to continue the present course in which PEH is a subset of general pediatrics. A fourth option, not incompatible with any of the others would be to form an International Society for Children’s Health and the Environment with credentialing authority (Lanphear et al. 2006).

Pediatric environmental health is still an emerging discipline (Landrigan et al. 2004; Woolf and Quang 2000). Yet the origins of environmental medicine are to be found in the writings of Hippocrates, who advised all physicians to consider the influence of the environment on their patients’ health. The need for a trained and properly credentialed professional workforce in pediatric environmental medicine is a national priority.

## Addendum

At the end of “Program Description,” a new section, “Conclusion of funding,” has been added.

## Figures and Tables

**Table 1:** Demographic characteristics of PEH fellows, United States, 2001–2007.

Characteristic	No. (%)
Age [years (mean ± SD)]	37 ± 6
Sex (male)	8 (47)
Race/ethnicity	
White	8 (47)
African American	1 (6)
Latino	2 (12)
Asian	6 (35)

**Table 2:** Current positions and research productivity of current PEH fellows and program graduates.

	Training complete (*n* = 13)	Training incomplete (*n* = 4)
On full-time academic faculty	9 (69)	NA
Senior government scientist	1(8)	
Scientist with national environmental organization	1(8)	
No. who have been a first author	10 (77)	2 (50)
Average no. of first-authored papers per fellow	2.1 ± 0.9 (1–3)	0.3 ± 0.5 (0–1)
PI on federal grants	5 (38)	0 (0)
PI on foundation grants	2 (15)	0 (0)

Abbreviations: NA, not applicable; PI, principal investigator. Values are no. (%) or mean ± SD (range).

**Table 3:** Predictors of publishing one or more papers among PEH fellows [no. (%)].

	≥1 paper (*n* = 10)	< 1 paper (*n* = 5)	*p-*Value
Age [years (mean ± SD)]	37.3 ± 5.7	37.6 ± 6.1	0.93
Male sex	5 (50)	2 (40)	0.71
White race	5 (50)	0 (0)	0.17^a^
Wrote extramural grant during fellowship	5 (50)	2 (40)	0.71
Remains affiliated with fellowship training site	7 (70)	0 (0)	0.04^a^*

a Exact estimation.

* Significant at the 0.05 level.

**Table 4:** Predictors of having a federal or nonfederal research grant as principal investigator among PEH fellows.

	≥1 grant (*n* = 12)	< 1 grant (*n* = 3)	*p-*Value
Age (years)	37.6 ± 5.9	36.7 ± 5.5	0.81
Male sex	6 (50)	1 (33)	0.61
White race	4 (33)	1 (33)	NA
> 2 years fellowship training	6 (50)	3 (100)	0.23
Remains affiliated with fellowship training site	4 (33)	3 (100)	0.08
Percentage of fellowship time allocated to various activities			
Class work	17 ± 15	18 ± 20	0.86
Clinical practice	12 ± 8	33 ± 15	0.003
Clinical or research teaching	12 ± 8	10 ± 13	0.71
Conducting research	48 ± 17	30 ± 22	0.15
Wrote extramural grant during fellowship	6 (50)	1 (33)	0.61

NA, not applicable. Values are mean ± SD or no. (%).
